# Systematic identification of biochemical networks in cancer cells by functional pathway inference analysis

**DOI:** 10.1093/bioinformatics/btac769

**Published:** 2022-11-30

**Authors:** Irbaz I Badshah, Pedro R Cutillas

**Affiliations:** Centre for Genomics and Computational Biology, Barts Cancer Institute, Queen Mary University of London, London EC1M 6BQ, UK; Centre for Genomics and Computational Biology, Barts Cancer Institute, Queen Mary University of London, London EC1M 6BQ, UK

## Abstract

**Motivation:**

Pathway inference methods are important for annotating the genome, for providing insights into the mechanisms of biochemical processes and allow the discovery of signalling members and potential new drug targets. Here, we tested the hypothesis that genes with similar impact on cell viability across multiple cell lines belong to a common pathway, thus providing a conceptual basis for a pathway inference method based on correlated anti-proliferative gene properties.

**Methods:**

To test this concept, we used recently available large-scale RNAi screens to develop a method, termed functional pathway inference analysis (FPIA), to systemically identify correlated gene dependencies.

**Results:**

To assess FPIA, we initially focused on PI3K/AKT/MTOR signalling, a prototypic oncogenic pathway for which we have a good sense of ground truth. Dependencies for *AKT1*, *MTOR* and *PDPK1* were among the most correlated with those for *PIK3CA* (encoding PI3Kα), as returned by FPIA, whereas negative regulators of PI3K/AKT/MTOR signalling, such as *PTEN* were anti-correlated. Following FPIA, *MTOR*, *PIK3CA* and *PIK3CB* produced significantly greater correlations for genes in the PI3K-Akt pathway versus other pathways. Application of FPIA to two additional pathways (p53 and MAPK) returned expected associations (e.g. *MDM2* and *TP53BP1* for p53 and *MAPK1* and *BRAF* for MEK1). Over-representation analysis of FPIA-returned genes enriched the respective pathway, and FPIA restricted to specific tumour lineages uncovered cell type-specific networks. Overall, our study demonstrates the ability of FPIA to identify members of pro-survival biochemical pathways in cancer cells.

**Availability and implementation:**

FPIA is implemented in a new R package named ‘*cordial*’ freely available from https://github.com/CutillasLab/cordial.

**Supplementary information:**

Supplementary data are available at *Bioinformatics* online.

## 1 Introduction

Signal transduction pathways decode extracellular signals into appropriate biological responses, thereby regulating most cell processes. Components of signalling pathways form complex networks of biochemical reactions and are frequently dysregulated in diseases such as cancer, neurodegeneration and diabetes. Therefore, discovering new signalling pathway members is important to understand the mechanisms of cellular biological processes and to uncover new anti-cancer drug targets. Pathway inference methods used for this purpose are based on literature mining techniques (Prill *et al.*, 2011; Turei *et al.*, 2016), on protein–protein interaction analysis (Choobdar *et al.*, 2019; Singh *et al.*, 2008) or on the identification of co-correlated genes in transcriptomics data (van Dam *et al.*, 2018). More recently, kinase networks have been mapped using phosphoproteomic markers of kinase–kinase associations (Hijazi *et al.*, 2020; Terfve *et al.*, 2015).

Despite these advances, assigning genes to pathways and reconstructing cell type-specific networks is still challenging (Turei *et al.*, 2016). Experimental approaches for pathway inference do not take into account protein function, whilst literature mining methods are influenced by gene popularity and reagent availability. We postulated that genes belonging to the same pro-proliferative pathway would produce the same anti-proliferative phenotypes across cell models when such genes are perturbed. Thus, analysis of pairwise correlated anti-proliferative signals in systematic genetic perturbation screens should reveal components of signalling pathways that regulate cell survival and proliferation.

To test this concept, we developed a method named functional pathway inference analysis (FPIA), which uses gene dependency data (cell viability data as a function of genome-wide gene targeting) to reveal genes whose inhibition produces similar cell survival phenotypes across cell lines. This is distinct from previously published methods where the aim was to identify genetic or other perturbations correlated to gene dependencies (Park *et al.*, 2022). To evaluate the performance of FPIA, we chose to examine the Class 1A phosphoinositide 3-kinase (PI3K)-AKT signalling pathway as this is one of the most frequently dysregulated pathways in cancer (Bachman *et al.*, 2004; Boehm and Golub, 2015; Meyers *et al.*, 2017; Tsherniak *et al.*, 2017; Vanhaesebroeck *et al.*, 2010; Yu *et al.*, 2016). This pathway is well characterized (Geering *et al.*, 2007; Porta *et al.*, 2014); Class 1A PI3K consists of regulatory (*PIK3R1*, *PIK3R3* and *PIK3R3* isoforms) and catalytic (*PIK3CA*, *PIK3CB* and *PIK3CD*) subunits. When activated by upstream signals, such as those emanating from receptor tyrosine kinases (examples of which include EGFR and the insulin receptor), PI3K phosphorylates phosphatidylinositol 4,5 bisphosphate (PIP_2_) to produce phosphatidylinositol 3,4,5 trisphosphate (PIP_3_). This lipid second messenger nucleates protein complexes leading to the activation of an intracellular protein kinase cascade involving PDK1 (gene *PDPK1*), AKT1 and MTOR. The lipid phosphatase PTEN dephosphorylates PIP_3_, thus opposing and terminating PI3K signalling. The PI3K system therefore offers a system for which we have a good sense of ground truth and thus allows assessing the ability of FPIA to produce biologically meaningful results. FPIA was then further tested for its ability to return meaningful associations to p53 and MEK1.

## 2 Materials and methods

### 2.1 Data availability

FPIA utilized as input cancer dependency datasets from the Cancer Dependency Map (DepMap) project—a collaboration of the Broad Institute (Cambridge, MA, USA) and the Wellcome Sanger Institute (Hinxton, Cambridgeshire, UK). Data produced by the DepMap project are made publicly available under a Creative Commons licence and are accessible through the portal (Boehm and Golub, 2015; Corsello *et al.*, 2020; Meyers *et al.*, 2017; Tsherniak *et al.*, 2017; Yu *et al.*, 2016). Here, we used the ‘D2_Achilles_gene_dep_scores’ dataset containing 711 cell lines (in rows), 16 810 genes (in columns) and 31 tumour lineages (Marcotte *et al.*, 2016; McDonald *et al.*, 2017; McFarland *et al.*, 2018; Tsherniak *et al.*, 2017); analysis of the *PIK3CD* gene was not included as it was absent in the dataset. The associated cell line sample data included DepMap ID, cell line display name and lineage type with three further lineage subtypes. Data were manipulated using the R programming language (v4.1.2) in RStudio (v1.4.1717).

### 2.2 Correlation analysis function design

FPIA implemented Pearson correlation analysis to assess the similarity in dependency data via three principal functions of the ‘*cordial*’ package constructed herein: *cor_target_map()* and *cor_targets()* utilized *cor.test()* of the *stats* R package to produce correlations for a vector of targets or a single target respectively, and *cor_map()* used *pwcor()* of the *collapse* R package to generate correlations for an entire dataset. Calculated significance levels (*p*-values) were subjected to the Benjamini–Hochberg multiple testing adjustment method with *stats::p.adjust()* to control the false discovery rate calculating *q*-values; ‘*cordial*’ functions have the capacity to set the adjustment method (parameter *method*) which is passed to *stats::p.adjust()* (Benjamini and Hochberg, 1995). The general method applied was to firstly filter and subset input data; secondly, perform correlation analysis calculating *r*-values and *P*-values with multiple testing adjustment; thirdly, construct the output by transforming the correlation matrix into a long data format (*data.table*) to facilitate downstream analysis. The ‘*cordial*’ package was designed to be flexible so as not to be dependent on the input data and as such it can be used for correlation analysis of datasets beyond the molecular/genetic screens studied in this research.

Correlation analysis was performed in a vectorized and parallelized manner with the *future* package to provide the parallel backend, and vectorized mapping functions from the *furrr* package that process input in parallel (Vaughan and Dancho, 2021). Prior to function usage, a multisession plan (*plan()*) was created that generates multiple R sessions which resolve futures asynchronously (in parallel) in separate R sessions equal to the number of physical processor cores; for convenience, this process has been incorporated into the ‘*cordial*’ function *start_parallel()*. The input supplied was split, distributed to the workers and returned to the main R session to be combined once all chunks have been processed. This method of spawning multiple background R sessions ensures compatibility across all operating systems since the alternative of forking processes is not supported by Windows.

The function arguments consisted of an input dataset (parameter *dataset*) containing values which may also include associated metadata, or alternatively an optional dataset can additionally be supplied that may hold the metadata instead (parameter *metadata*). The data structure for both inputs is a *data.table* as this allowed for the subsequent efficient filtering of rows and subsetting of columns. Filtering (parameter *filter_rows*) was programmed by taking the input and a named *list()*, whereby names specify column names to filter on, and performing a cross-join. If the dataset required subsetting to limit the correlations to be made (or omit non-numeric value columns) then a vector of column names can be provided (parameter *select_cols*). The resultant correlations of *cor_map()* are symmetrical, specifically pairwise correlations are produced between all combinations of the columns specified (parameter *select_cols*). Unlike *cor_map()*, *cor_targets()* takes an additional argument of a single target column name (parameter *target*). The function *cor_target_map()* calls *cor_targets()* internally which is mapped in parallel with *future_map()* to the argument of a vector of column names. In contrast with *cor_map()*, *cor_target_map()* creates non-symmetrical correlations, that is for each specified target column (parameter *target*), pairwise correlations are produced with the columns specified (parameter *select_cols*).

### 2.3 Statistical analyses

Statistical methods (from the package *stats()*) were used to determine the statistical significance of results and check assumptions were met. These included: Shapiro–Wilk test for normality (*shapiro.test()*), F-test for homogeneity in variances (*var.test()*) and Welch two-sample unpaired *t*-test (*t.test()*). Fisher *z*-transformation (*psych::fisherz*) of the Pearson correlation coefficient *r* was used prior to testing of statistical significance for the population correlation coefficient *ρ* to overcome the skewed distribution observed as the sample correlation coefficient *r* nears ±1.

### 2.4 Hierarchical cluster analysis

Hierarchical cluster analysis was performed to assess gene relationships. Dissimilarities of Euclidean distances of *r*-values from significantly correlated genes following Pearson correlation, which included self-correlations, were calculated with *dist()* and subsequently used for clustering. The linkage method applied Ward's minimal increase of sum-of-squares agglomeration method, corresponding to *hclust(…, method = ‘ward.D2’)*, which minimized within-cluster variance (Murtagh and Legendre, 2014). A clustered heatmap was subsequently created with *Heatmap()* of the *ComplexHeatmap* package (Gu *et al.*, 2016).

### 2.5 Network analysis

Network analysis of the gene correlations was conducted using the *igraph* package and the *graph_from_data_frame()* function (Csardi and Nepusz, 2006). The edge weight attribute (*E(graph)$weight*) was assigned as absolute *r*-value; the edge colour attribute (*E(graph)$color*) was assigned to the direction of the correlation (positive/negative); the edge width parameter (*edge.width*) was assigned inside the call to the plotting function *plot.igraph()* as −log_10_(*q*-value). The vertex (node) colour attribute (*V(graph)$color*) was assigned to being a target gene. The vertex size parameter (*vertex.size*) was assigned in *plot.igraph()* as weighted vertex degree with *strength()*. Network groups were calculated in the *plot.igraph()* parameter (*mark.groups*) as connected components (*groups(components(graph))*). The layout of the network graph (*plot.igraph(…, layout = layout_with_fr, …)*) was the Fruchterman and Reingold force-directed layout where larger weight values (herein absolute *r*-value) produced shorter edges (Fruchterman and Reingold, 1991). Self-correlations were omitted to avoid self-loops. The analyses performed were *degree()* (the number of adjacent edges of a vertex) and *betweenness()* (the number of shortest paths through a vertex) (Csardi and Nepusz, 2006).

### 2.6 Gene ontology over-representation analysis

The output from *cor_target_map()* was studied by Term Enrichment Analysis of genes correlated to the target gene in Reactome (Yu and He, 2016) as previously described in Gerdes *et al.* (2021) (https://github.com/CutillasLab/Term-Enrichment-Analysis); the false discovery rate was controlled for by calculating *q*-values with the Benjamini–Hochberg method (Hochberg and Benjamini, 1990).

## 3 Results and discussion

### 3.1 FPIA identifies core signalling networks

#### 3.1.1 Identification of gene-specific canonical signalling pathways

**Fig. 1.**
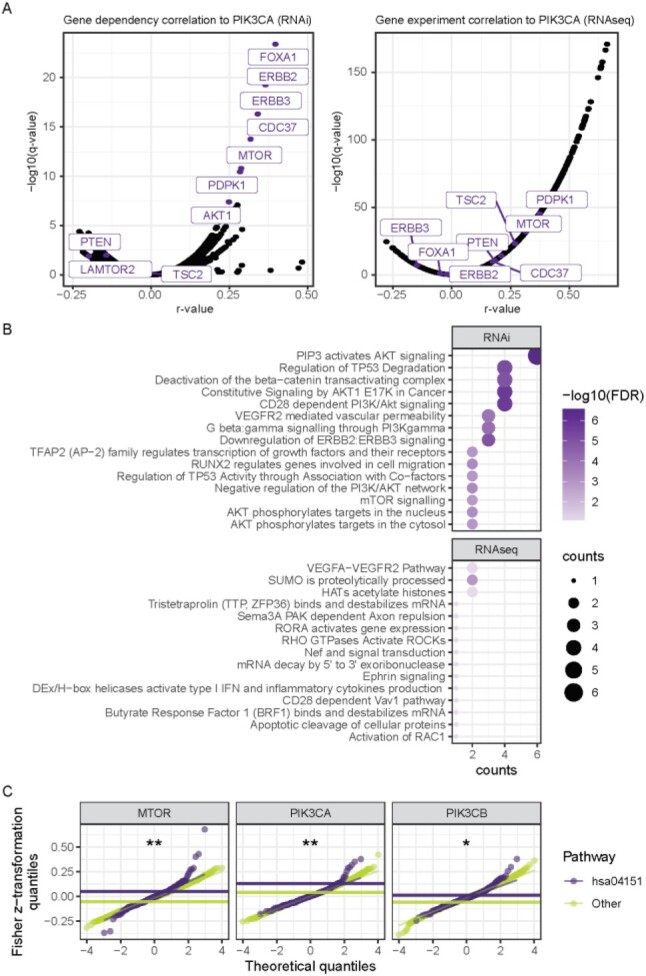
Functional pathway inference analysis identifies components of proliferative signalling pathways. (**A**) Illustration of the FPIA approach, showing gene dependencies (left) or gene expression (RNAseq, right) significantly associated to those for *PIK3CA* across cancer cell lines. (**B**) Over-representation analysis of *PIK3CA* significant correlations from RNAi (top) and RNAseq (bottom) data. (**C**) Quantile–quantile plots of FPIA performed on RNAi data following Fisher *z*-transformation of *r*-value in which the target genes (shown in graph headers) that possessed means (shown as horizontal lines) of significant correlations belonging to the pathway hsa04151 as being significantly greater than those of other pathways, ranked by *t*-test *P*-value; ** *P *<* *0.01, * *P *<* *0.05

To assess the accuracy of FPIA as an unbiased pathway inference tool, we applied it to a set of 25 human target genes canonically associated with PI3K signalling as given by the KEGG pathways hsa04151: PI3K-Akt signalling pathway, or hsa04070: phosphatidylinositol signalling system. The dependencies of these 25 canonical PI3K genes across the 711 cell lines were correlated to the dependencies of the other 16 810 genes in the RNAi dataset in a pairwise manner using the *cor_target_map()* function in the ‘*cordial*’ package developed as part of this work. Figure 1A demonstrates proof of principle for gene dependencies associated with those for the *PIK3CA* gene, which codes for the p110α catalytic subunit of PI3K. Comparing this to RNAseq data as in previous work (van Dam *et al.*, 2018), FPIA using dependencies from DepMap RNAi screens was able to identify the most strongly correlated genes as being significant, whereas the RNAseq-based analysis failed to do so (Fig. 1A). Furthermore, this was reinforced by over-representation analysis of the top 25 *PIK3CA* significant correlations for each method, in which FPIA with RNAi, but not RNAseq, returned significant terms closely related to PI3K-AKT signalling (Fig. 1B).

FPIA found 13 725 genes in the dataset (81.6%) that were significantly correlated (*q *<* *0.05) to target genes, of which 300 were in hsa04151 from the 342 hsa04151 genes present in the RNAi dataset, producing a pathway coverage of 87.7% (Supplementary Fig. S1). The target gene with the greatest number of significant correlations in hsa04151 was *INPPL1* (91) followed by *INPP5E* (75), and this was transposed for significant correlations of genes corresponding to other pathways (Supplementary Fig. S1). Both target genes belong to hsa04070, whereas the highest ranked for hsa04151 were *PIK3R1* (71) and *PDPK1* (69). *INPPL1* and *INPP5E* are involved in PI-associated signalling and thus can only indirectly influence AKT signalling by manipulating the pool of PI lipid substrates, whilst *PIK3R1* encodes a regulatory subunit of PI3K (Vanhaesebroeck *et al.*, 2010). Such gene products are consequently capable of exerting influence across multiple PI3K members within the pathway. The observed ranking is therefore consistent with the nature of molecular interactions of PI3K signalling nodes. To determine if PI3K target genes were more correlated to PI3K pathway members than to other pathways, we carried out a Pearson correlation *r*-value to Fisher *z*-transformation of significant correlations (*q*-value < 0.05) followed by *t*-test of hsa04151 genes versus genes of all other pathways. This analysis revealed six target genes where the means of the significant correlations that belong to the pathway were significantly different to those of other pathways. Of these, *MTOR*, *PIK3CA* and *PIK3CB* exhibited means that were significantly greater for correlations to genes in hsa04151 than other pathways (Fig. 1C). Together, these results demonstrate that from only a small sample of target genes, FPIA is a useful method that capably identifies the majority of a canonical signalling pathway.

**Fig. 2.**
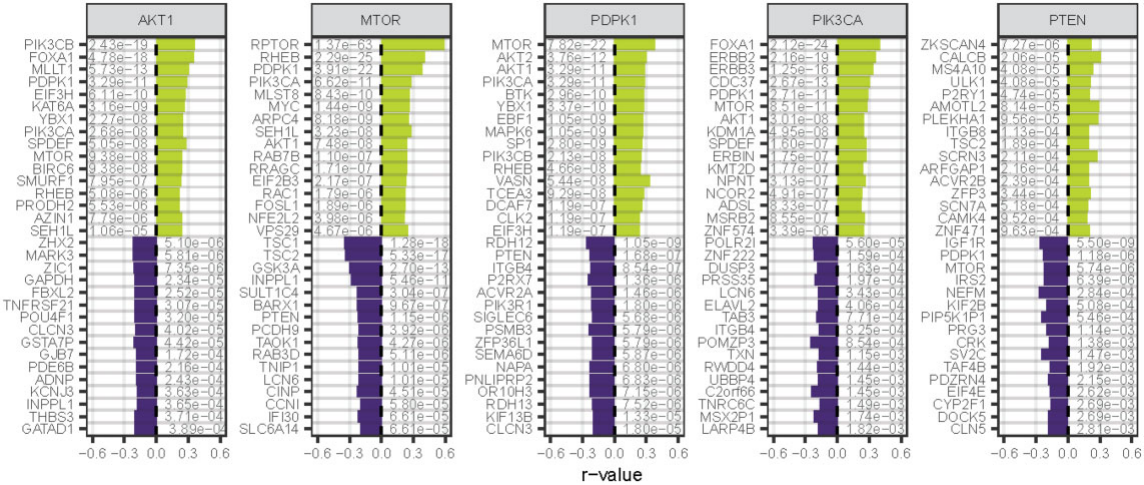
FPIA identifies components associated to canonical PI3K signalling members. Topmost positively and negatively significantly correlated gene dependencies (in y-axes) to those of a subset of PI3K-associated target genes (shown in graph headers). Data ranked by *q*-value (shown for all genes as labels); self-correlations omitted

To test the performance of FPIA in returning biologically meaningful associations further, we assessed the output of the approach when querying individual PI3K/AKT pathway members (Figs 1A and 2, Supplementary Fig. S2). In agreement with the canonical understanding of PI3K signalling (Geering *et al.*, 2007; Hutchinson *et al.*, 2004; Manning and Cantley, 2007; Porta *et al.*, 2014), FPIA showed that dependency to *PIK3CA* was positively correlated with dependencies to downstream kinases such as *AKT1*, *MTOR* and *PDPK1*, the transcription factor *FOXA1*, and the upstream PI3K activators *ERBB2* and *ERBB3*, as well as the ERBB2 regulator *ERBIN* (Figs 1A and 2, Supplementary Fig. S2). Furthermore, the results were coherent with one another, as illustrated by *AKT1* and *MTOR* which were positively correlated with each other regardless of which gene was used as the target. This also applied to correlated genes that were not themselves queried such as *ERBB2*, *ERBB3* and *SPDEF* that were positively correlated with *PIK3CA*, *AKT1*, *MTOR* and *PDPK1*, which in turn were congruently positively correlated with each other. Similarly, dependencies to *MTOR* correlated to those for *MTOR* binding partners *RAPTOR* and *RHEB*. Additionally, dependencies to known negative regulators of the pathway, such as *PTEN, TSC1* and *TSC2*, reversely correlated with those to *MTOR* whilst being positively correlated with each other. Hierarchical cluster analysis revealed clear grouping that demarcated the direction of gene correlations (positive or negative) which was homogenous for all key target genes (Supplementary Fig. S6). Furthermore, it demonstrated *PIK3CA* and *AKT1* to be less dissimilar than *MTOR* and *PDPK1*, causing the clustering of two groups, which followed the nature of their known interactions (Supplementary Fig. S6). Overall, these results show that FPIA is able to return biologically coherent results and reproduce components associated to a canonical signalling pathway with high sensitivity.

Beyond the ability to output known associations, significant gene correlations that were unknown from our canonical understanding of the target gene signalling network were also produced. *PIK3CA* dependencies across cells were positively correlated with those of *ZNF574* (Fig. 2, Supplementary Fig. S2). While no experimental evidence in the literature exists for a functional interaction between *ZNF574* and PI3K signalling (a search in PubMed returned zero publications), there is support to suggest that this may be a true observation as both have similar mutation patterns in cancer (Ahn *et al.*, 2016; Arafeh and Samuels, 2019). Additionally, *ZNF574* is differentially upregulated in early-onset colorectal cancer versus late-onset, and *PIK3CA* mutations are elevated in early-onset hypermutated colorectal cancer cases (Ahn *et al.*, 2016; Berg *et al.*, 2010). The approach similarly returned associations of genes that are not part of the canonical PI3K/AKT pathway, but for which some experimental evidence exists for their biological relevance, such as with the kinase MAPKAPK2 found to be downstream of the PI3K/MTOR axis (Herranz *et al.*, 2015). These results illustrate the potential of FPIA to discover new components of pro-proliferative biochemical pathways and provide novel insights into signalling networks.

#### 3.1.2 Identification of tumour lineage-specific PI3K networks

**Fig. 3.**
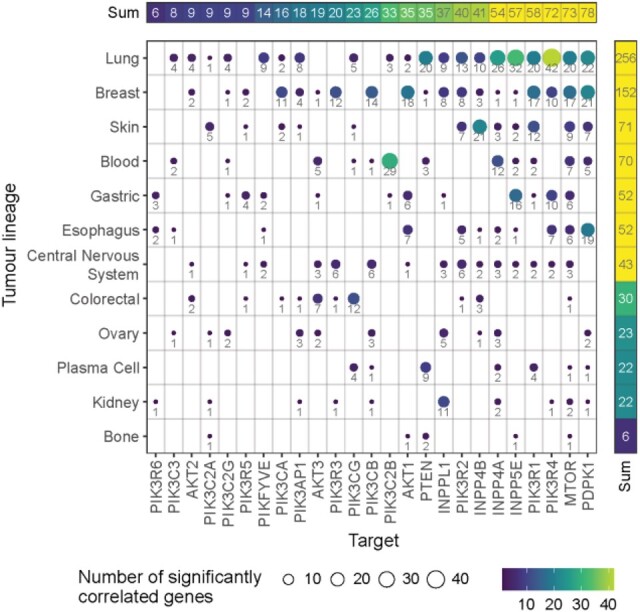
FPIA allows identifying tumour lineage-specific signalling. PI3K-associated target genes and their significant correlations in a predefined subset of genes belonging to KEGG pathway hsa04151 (in addition to the target gene set) for individual tumour lineages within the combined RNAi DepMap dataset (*q *<* *0.05). Data ranked by counts (labelled) of significant correlations; self-correlations omitted

To obtain an insight into the distinct nature of signalling cascades in a tumour-specific context, we applied FPIA to the canonical PI3K gene set (KEGG pathway hsa04151) within individual tumour lineages. Of the 31 lineages represented in the dataset, 12 had a sufficient number of cell lines without missing values (*n *>* *4) for analysis. Although this represents a restriction, it is of the input dataset rather than the method and therefore will improve as more cancer models are studied. The three tumour lineages that cumulatively covered the greatest number of significantly correlated (*q *<* *0.05) genes in hsa04151 were lung, breast and skin (Fig. 3). This can be partly explained by those lineages possessing a larger proportion of cell lines which facilitated the generation of statistical significance. Nevertheless, the central nervous system lineage with the third largest number of cell lines only ranked seventh. The target gene with the most significant correlations in the lung lineage was *PIK3R4* (42), whereas in the breast it was *PDPK1* (21), whilst in skin it was *INPP4B* (21). The three topmost target genes ranked by number of significantly correlated genes were *PDPK1*, *MTOR* and *PIK3R4*, which contrasted from the gene-specific analysis in hsa04151 across all cell lines (Supplementary Fig. S1). Moreover, the tumour-specific analysis revealed the prominence of each target gene in individual tumours as well as the relative influence of the pathway for each tumour type.

**Fig. 4.**
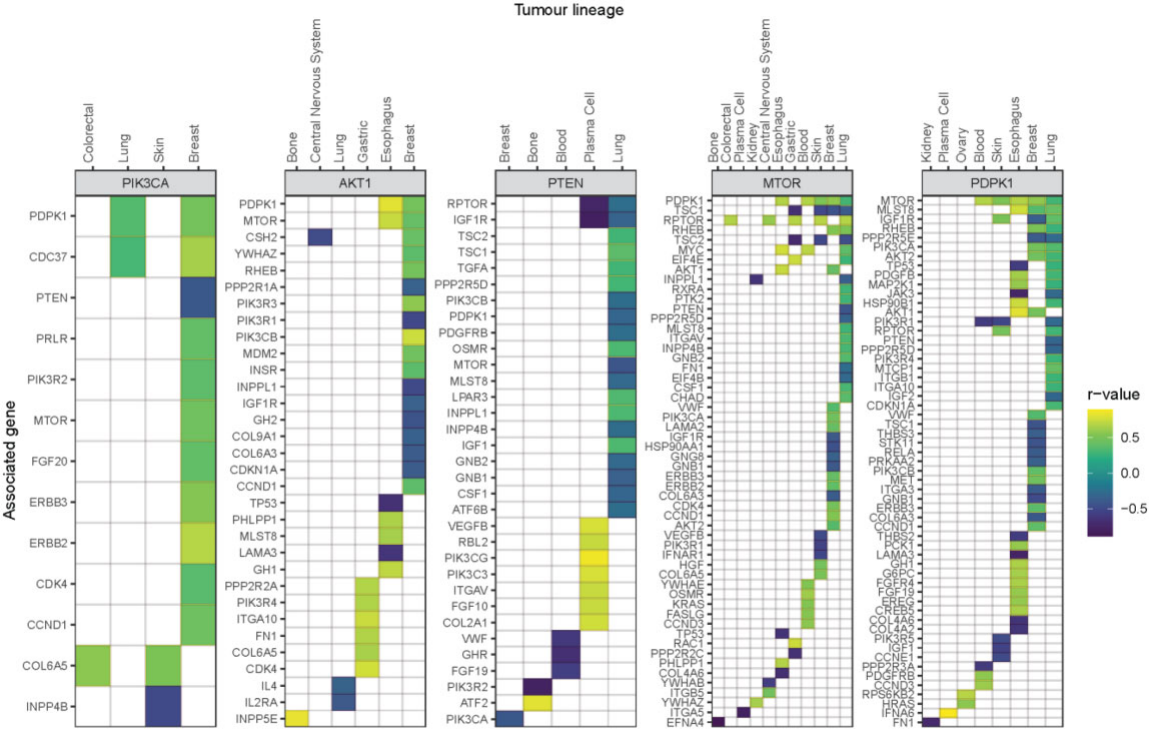
FPIA identifies correlated gene dependencies in hsa04151 within individual tumour lineages. Significantly correlated (*q *<* *0.05) gene dependencies in KEGG pathway hsa04151 for each PI3K-associated target gene (shown in graph headers) within individual tumour lineages. Correlated genes are ranked by counts in lineages, and tumour lineages ranked by counts of significant correlations; self-correlations omitted

To reveal a core PI3K network, we investigated the extent of conservation in cancer dependency in multiple lineages with pathway members possessing a similar dependency profile (Fig. 4, Supplementary Fig. S3). These findings illustrate that certain genes associate in a ubiquitous manner and form part of a canonical (or core) network. Whereas, for others their functional interacting partners are specific to a given tissue or precise conditions, as observed with *COL6A5* which was significantly positively correlated with *PIK3CA* in colorectal cancer (Fig. 4, Supplementary Fig. S3). *COL6A5* belongs to the collagen family and is a constituent of the extracellular matrix, where mutations are associated with colorectal cancer (Kim *et al.*, 2021). These results are consistent with a recent study that used weighted correlation network analysis in oesophageal squamous cell carcinoma tissues to identify an association between collagen family members and the PI3K/AKT/MTOR pathway (Li *et al.*, 2019). Thus, results from FPIA are aligned with the literature and have the potential to uncover core signalling networks as well as new cell type-specific pathway members.

To investigate which lineages each target gene featured in, and the magnitude of their involvement, tumour lineages were ranked by counts of significant gene correlations. *MTOR* dependency was associated with other gene dependencies in 11 out of 12 lineages, with the largest number of associations being in lung cancers (Fig. 4, Supplementary Fig. S3). In contrast, *PIK3CA* had significant dependency correlations in four lineages, of which breast held the most, followed by skin, lung and colorectal. Interestingly, consistent with these data, *PIK3CA* mutations are frequently found in breast cancers with 20–32% of tumours showing mutations in this gene, second only to endometrial cancers with 24–46% (Arafeh and Samuels, 2019). Also in line with our findings, *PIK3CA* mutation frequency in colorectal cancers is 13–28%, placing it as the fifth most frequently mutated gene in this malignancy (Arafeh and Samuels, 2019). Overall, our data show that FPIA may be used to identify the various cascades within a network that are likely to be involved in a given tumour type, thereby revealing sub-networks of pro-proliferative pathways and helping to rationalize differences in response to cancer treatment across tumour types.

### 3.2 Network analysis of FPIA results identifies central nodes

**Fig. 5.**
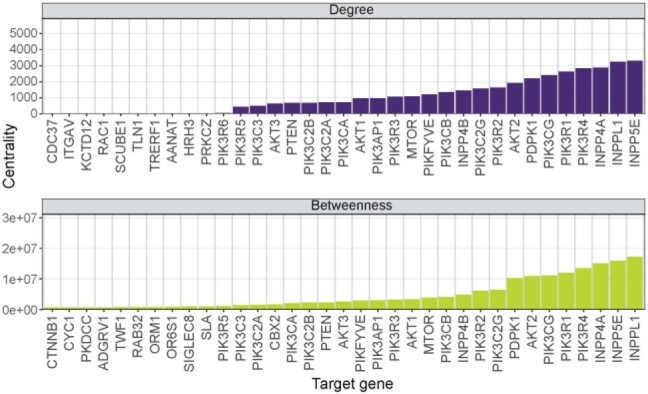
Network analysis of FPIA results identifies central nodes in the PI3K signalling network. Degree and betweenness centrality of the top-ranked vertices following FPIA of PI3K-associated target genes across all cancer cell lines, limited to significant dependency correlations (*q *<* *0.05). Data ranked by centrality; self-correlations omitted

To better interpret the relationships between signalling constituents of specific gene targets returned as significant by FPIA, we integrated these interactions in a network graph. In order to visualize the network and reveal its structure, it was simplified to display only the topmost significant correlations for each target gene (Supplementary Fig. S4). In agreement with the canonical understanding of the pathway, and with our previous results shown above, *PIK3CA*, *AKT1*, *MTOR* and *PDPK1* clustered most closely. To reveal which genes were the most influential, we calculated node degree centrality on the full network, whilst betweenness centrality was used to reveal vertices important in the flow of the network. The vertex with the largest betweenness centrality was *INPPL1*, mirroring the rank previously ascertained in Figure 1 (Fig. 5). *PIK3R1* ranked uppermost of the PI3K regulatory subunits, whilst *PIK3CG* ranked highest among the catalytic subunits. *INPPL1* represents a counterbalance to *PIK3CA*-generated PIP_3_, enabling its redistribution along a path of further phosphatase-mediated metabolism. Unlike *PTEN* which reverses the lipid product back into a substrate for *PIK3CA*, the action of *INPPL1* effectively sequesters the substrate from *PIK3CA*, thereby limiting the impact of *PIK3CA* action. Therefore, *INPPL1* is of vital importance in signal transduction in the PI3K/AKT network. The genes identified with a higher betweenness represented those that featured on a greater number of shortest paths. The significance of this being that they are instrumental in enabling movement through the signalling pathway and could potentially be critical cancer vulnerabilities to exploit. The method of ranking vertices according to measures of centrality allowed the determination of the most influential genes in the common pathway agnostic of intra-pathway prior knowledge. This focuses them for further study by pharmaceutical targeting, or for novel associations by corroboration *in vitro*.

### 3.3 FPIA identifies components of p53 and MAPK pathways

To determine whether FPIA can return meaningful associations for genes other than those in the PI3K/AKT/MTOR pathway, we applied the method to p53 (gene name *TP53*) and MEK1 (gene name *MAP2K1*, a member of the MAPK pathway). Genes that positively or negatively regulate p53, including *MDM2*, *MDM4*, *TP53BP1* and *CHEK2*, were the most significantly associated genes with *TP53* (Supplementary Fig. S5A). Similarly, the most significantly associated with genes to *MAP2K1* include upstream regulators of this kinase (*BRAF* and *STK38*) as well as downstream effectors [*MAPK1* (ERK2), *RPS6K1* (RSK) and *MAPK6*, *EIF2AK2* and *CDK19*] (Supplementary Fig. S5B). Term over-representation analysis showed an enrichment of TP53 and MAPKK activity pathways in *TP53* and *MAP2K1*, respectively (Supplementary Fig. S5A and B). In addition to these known members of p53 and MAPK axes, other genes with hitherto unknown roles in these pathways were also identified, highlighting the potential of FPIA for discovering new pathway members.

## 4 Conclusion

This work tested the hypothesis that genes with similar impact on cell viability across multiple cell lines belong to a common pathway, thus implying that the correlation of anti-cancer genetic dependencies may be used for biological pathway inference and for the annotation of genes with functional information. To test this idea, we developed a method termed FPIA to systemically determine correlated gene dependencies from large-scale gene silencing screens. Our gene-specific analysis revealed significant gene correlations within canonical signalling cascades with the potential to identify novel interactions. This was evident from inspection of PI3K, p53 and MEK1 correlated genes which returned the canonical pathway (*AKT1*, *MTOR*, *PDPK1*, *PTEN*, etc. for PI3K; ERK2, BRAF for MEK1; and MDM2 and TP53BP1 for p53), and by unbiased over-representation analysis in which the respectively associated pathways were enriched in the new unbiasedly identified network. Consequently, the FPIA method, and the associated *cor_target_map()* function of the ‘*cordial*’ package, provides insights into signalling pathways and may be used to uncover novel interactions in a global, genome-wide manner. A potential limitation of the method is that it relies on functional analysis of genes that impact cell viability and therefore it may only be applied to the discovery of members of pathways that regulate cell survival and the proliferative process. Nevertheless, since dysregulated cell proliferation is a key hallmark of cancer, and the aim of most anti-cancer drugs is to inhibit cell viability/proliferation, FPIA has the potential to advance our understanding of biochemical networks involved in cancer and to inform new therapeutic targets and approaches.

## Supplementary Material

btac769_Supplementary_Data
