# Supplementary material for: Systematic identification of biochemical networks in cancer cells by functional pathway inference analysis
Source: Bioinformatics. 2022 Nov 30;39(1):btac769. doi: 10.1093/bioinformatics/btac769 (PMC9805595; doi:10.1093/bioinformatics/btac769)
Supplement: btac769_Supplementary_Data [file btac769_supplementary_data.docx]

Systems biology

Systematic identification of biochemical networks in cancer cells by Functional Pathway Inference Analysis

Irbaz I. Badshah^1^ and Pedro R. Cutillas^1,*^

^1^Centre for Genomics and Computational Biology, Barts Cancer Institute, London, EC1M 6BQ, United Kingdom

^*^To whom correspondence should be addressed.


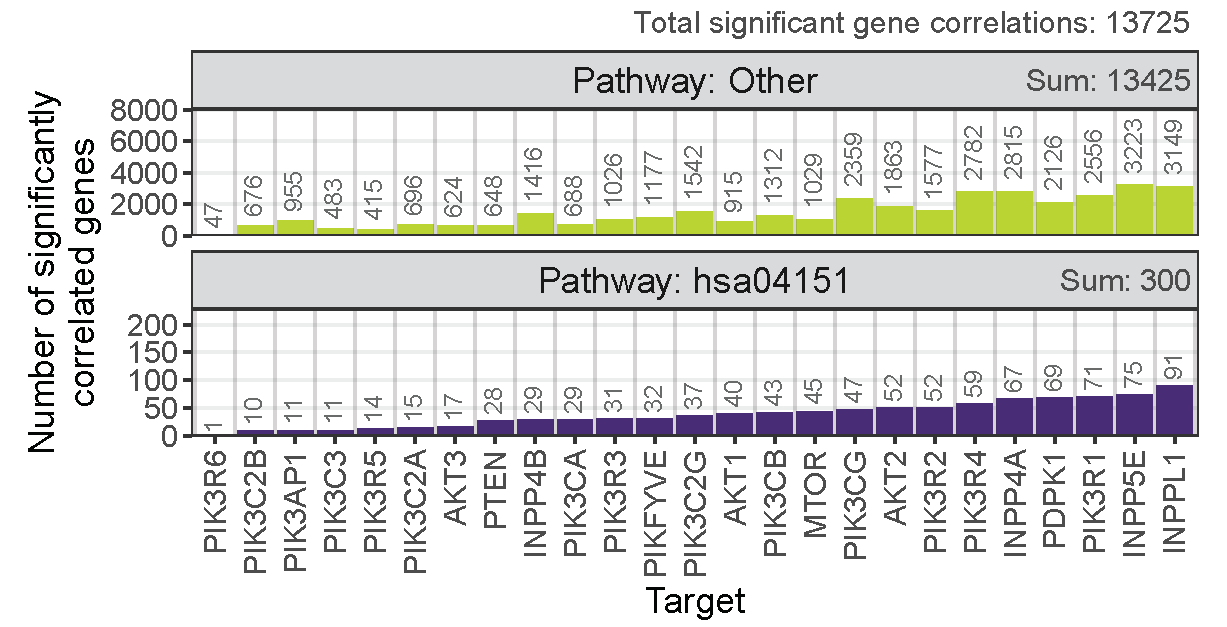


Figure S1. Coverage of significantly correlated genes in hsa04151.

A set of 25 PI3K-associated target genes subjected to Pearson correlation with Benjamini-Hochberg multiple testing adjustment method and the number of significant correlations (*q* < 0.05) in KEGG pathway hsa04151 and those external to the pathway; self-correlations omitted; target genes ranked by counts (shown in labels).


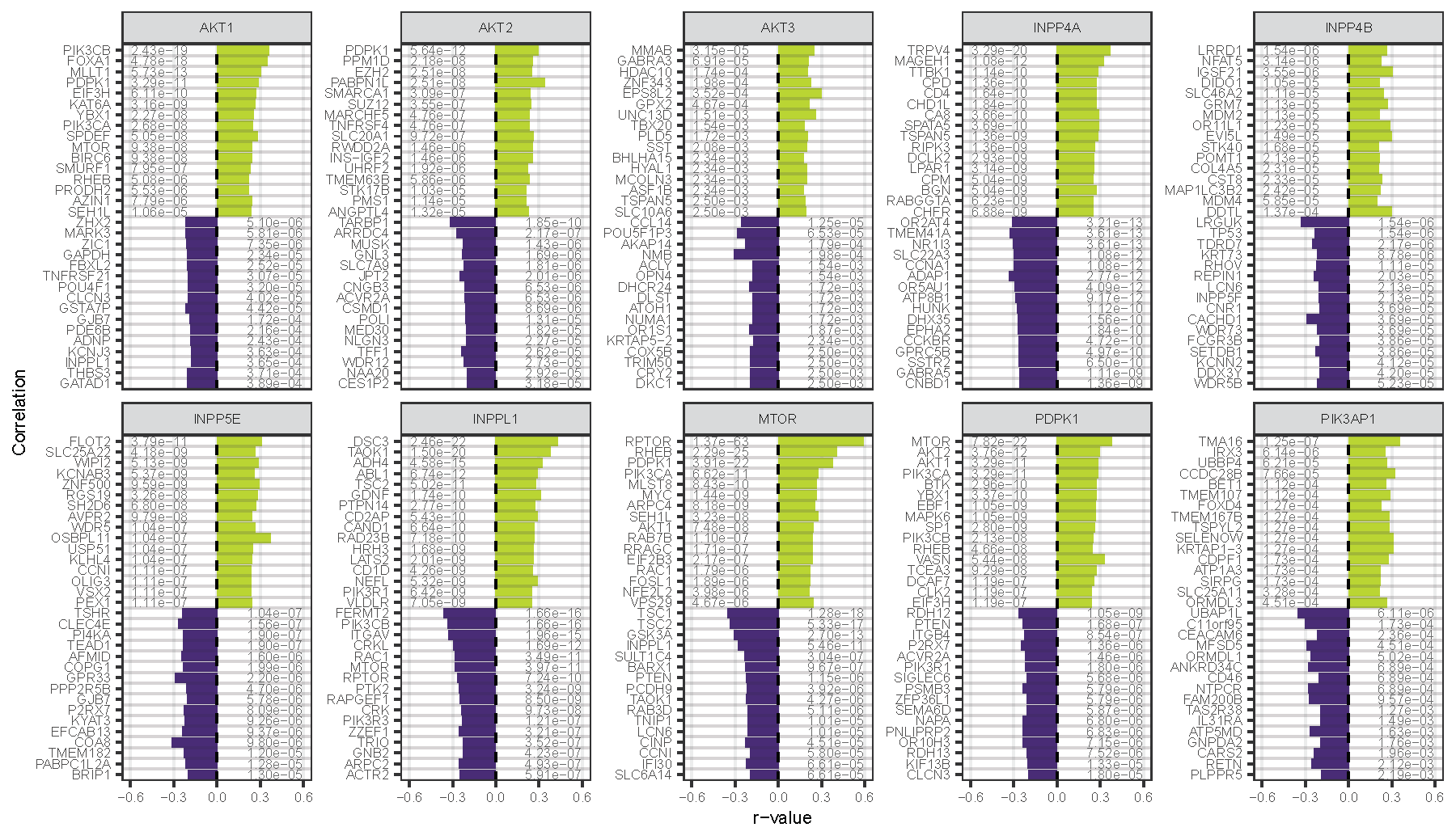


Figure S2. FPIA identifies components associated to canonical PI3K signaling members.

(Continues below)

Topmost positively and negatively significantly correlated gene dependencies (in y axes) to a set of PI3K-associated target genes (shown in graph headers). Data ranked by *q*-value (shown for all genes as labels); self-correlations omitted.


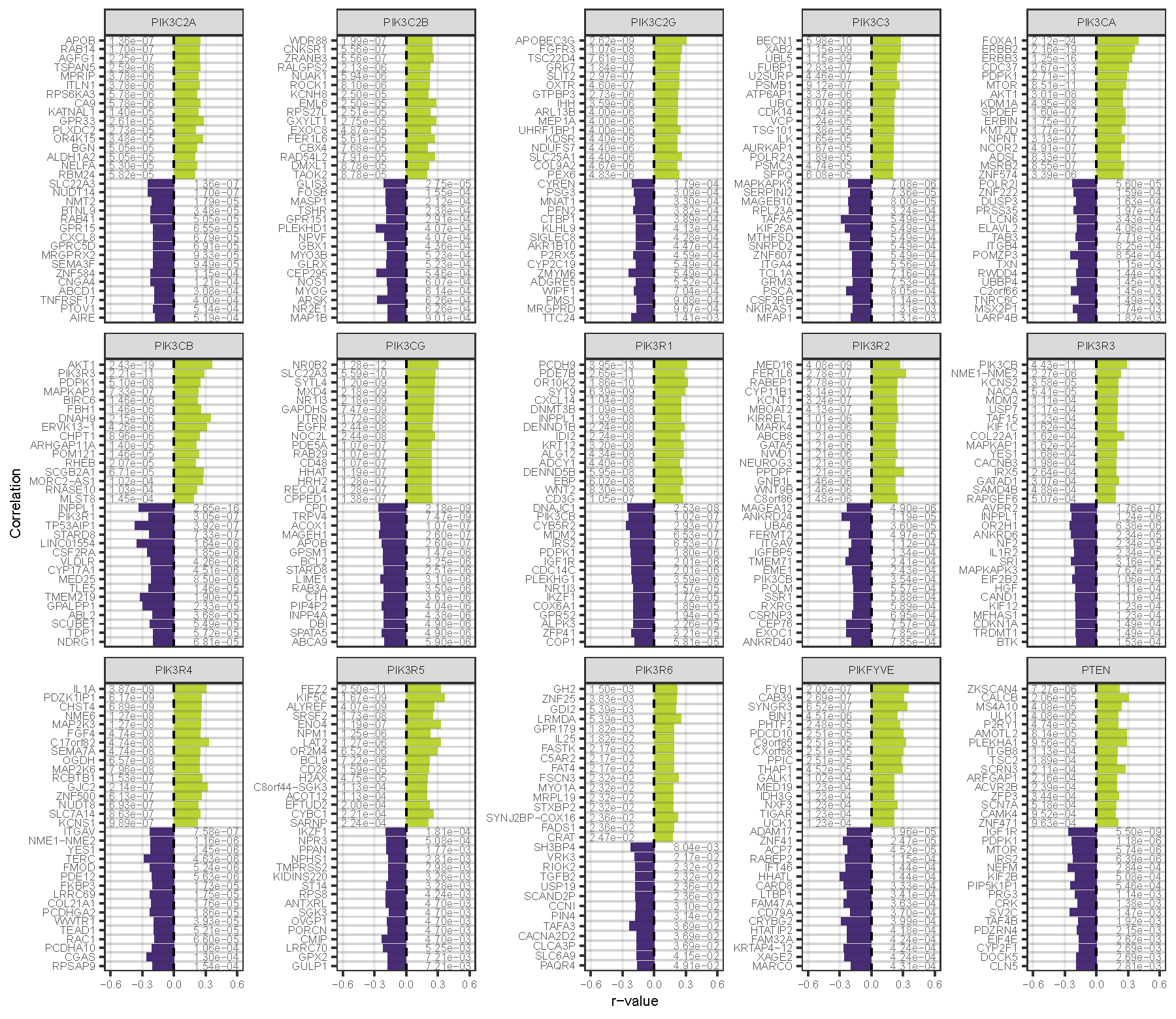


Figure S2. FPIA identifies components associated to canonical PI3K signaling members.

(Continued)

Topmost positively and negatively significantly correlated gene dependencies (in y axes) to a set of PI3K-associated target genes (shown in graph headers). Data ranked by *q*-value (shown for all genes as labels); self-correlations omitted.


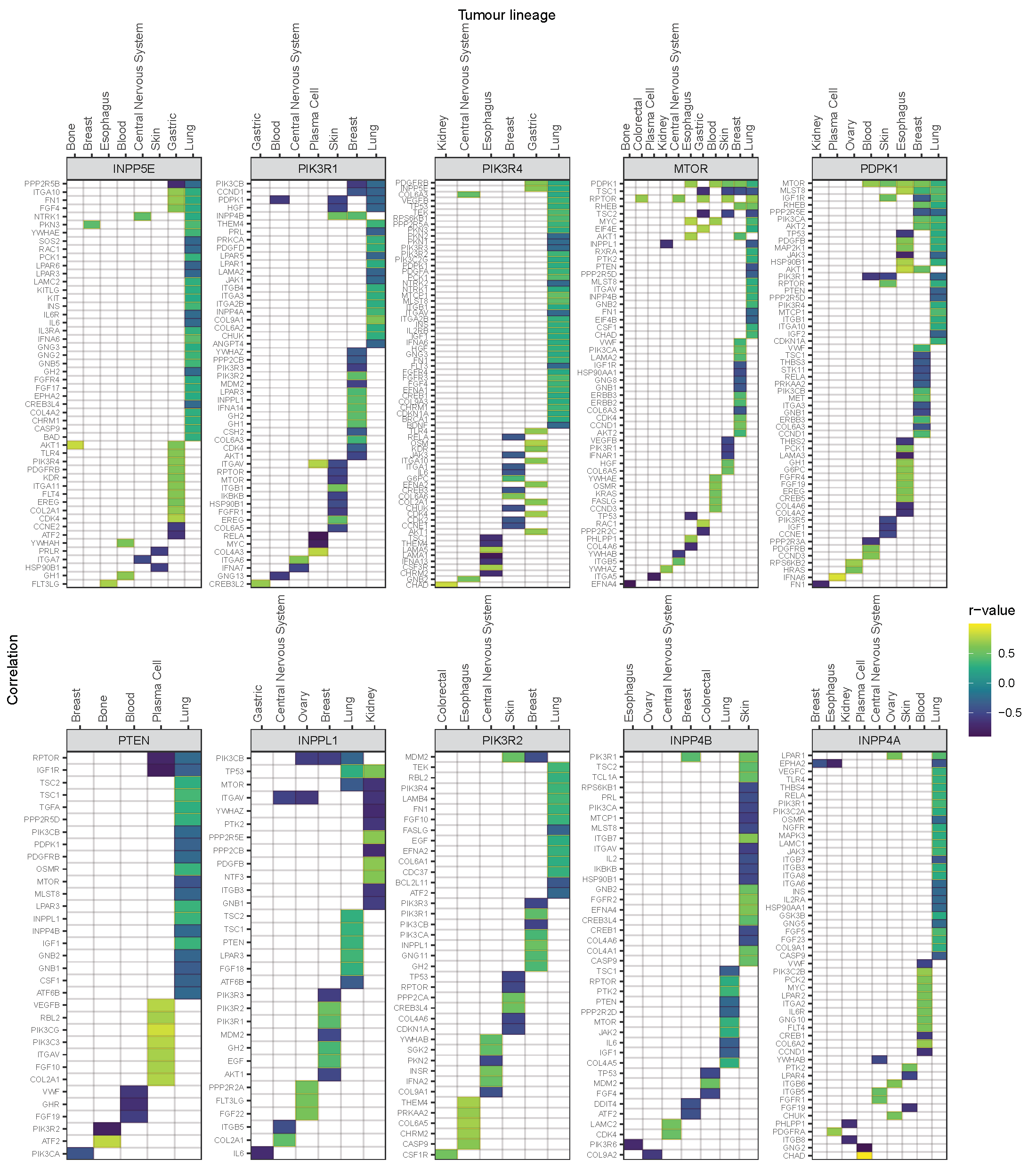


Figure S3. FPIA identifies correlated gene dependencies in hsa04151 within individual tumour lineages.

(Continues below)

Significantly correlated (*q* < 0.05) gene dependencies in KEGG pathway hsa04151 for each PI3K-associated target gene (shown in graph headers) within individual tumour lineages. Correlated genes are ranked by counts in lineages, and tumour lineages ranked by counts of significant correlations; self-correlations omitted.


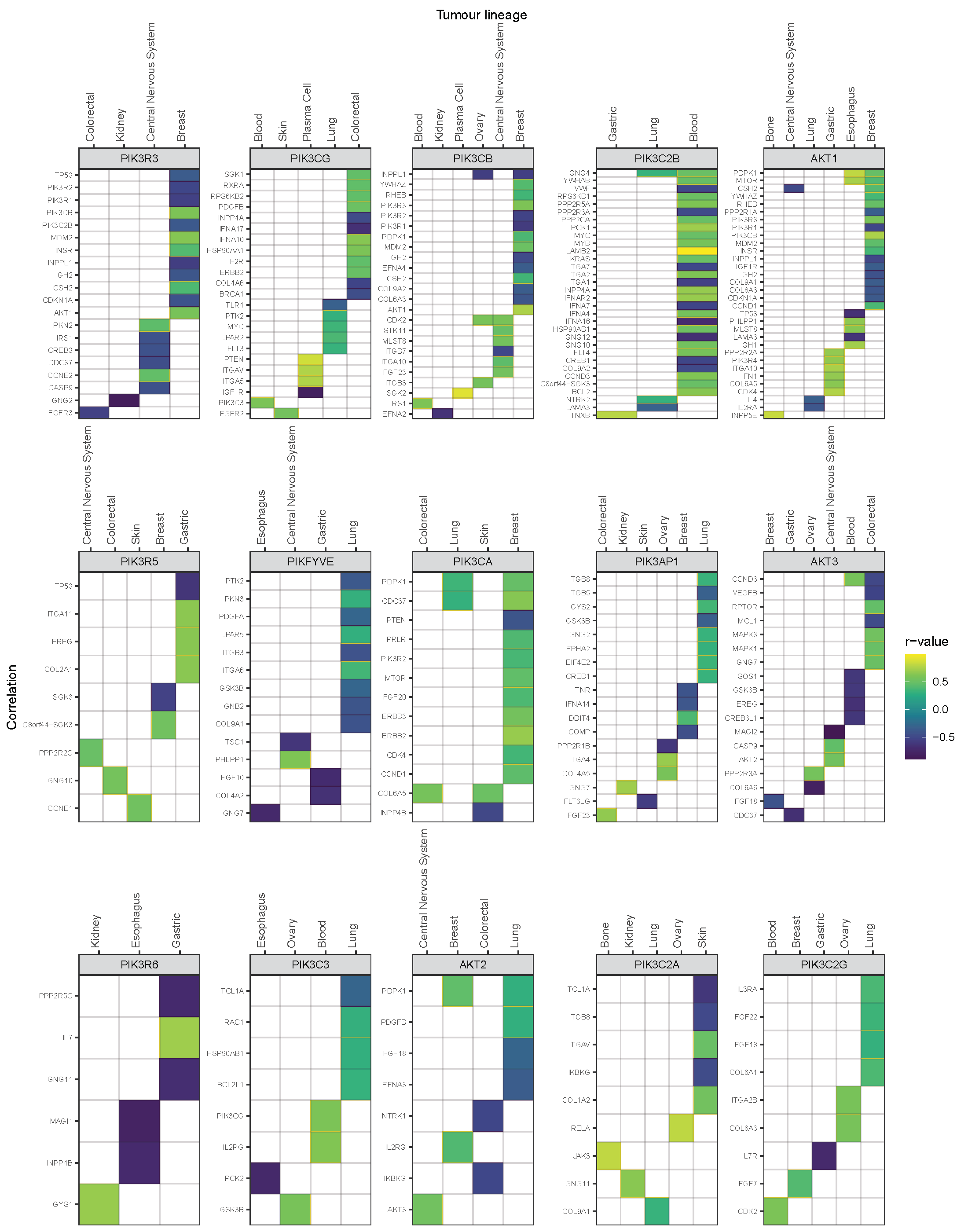


Figure S3. FPIA identifies correlated gene dependencies in hsa04151 within individual tumour lineages.

(Continued)

Significantly correlated (*q* < 0.05) gene dependencies in KEGG pathway hsa04151 for each PI3K-associated target gene (shown in graph headers) within individual tumour lineages. Correlated genes are ranked by counts in lineages, and tumour lineages ranked by counts of significant correlations; self-correlations omitted.


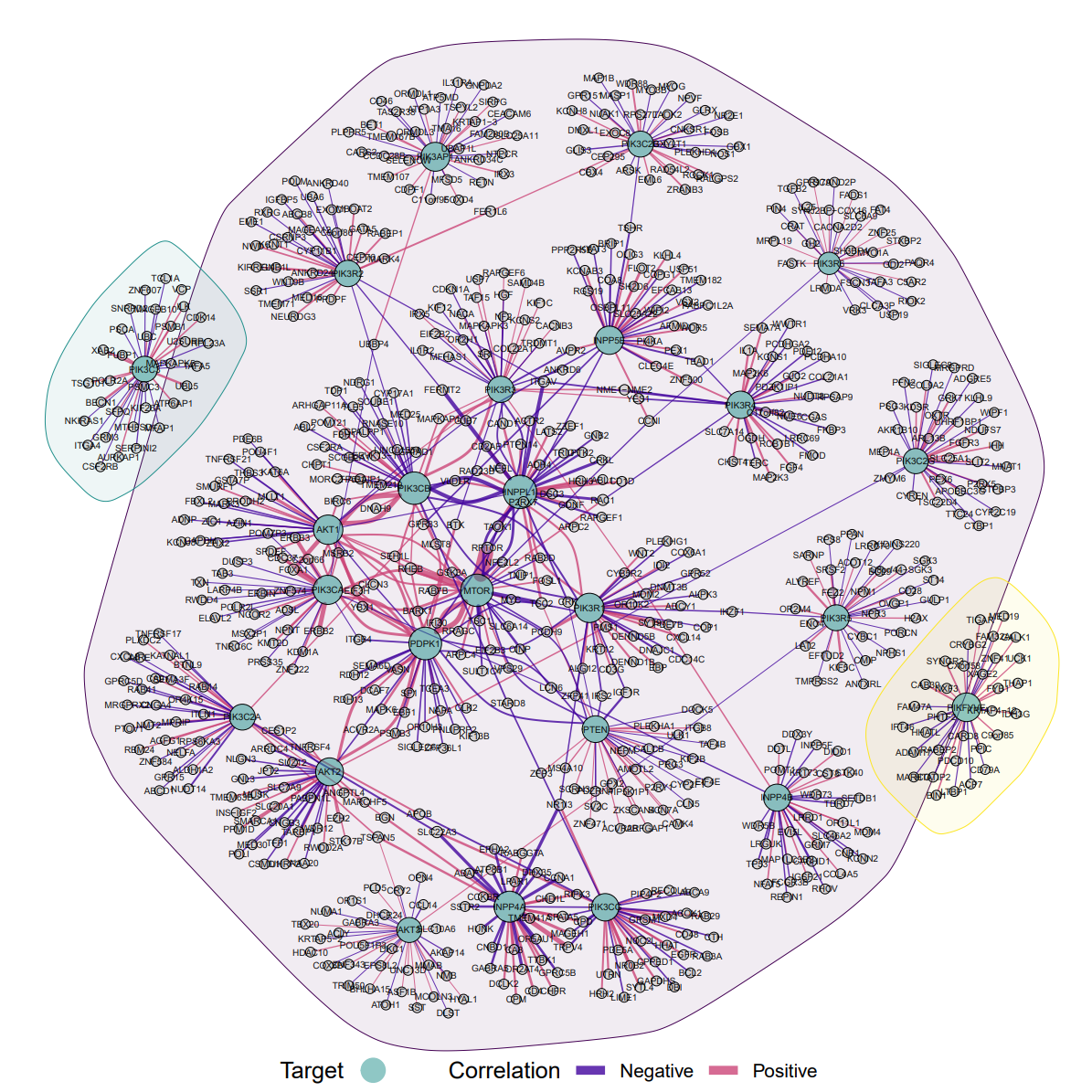
Figure S4. Network graph of the leading significant correlations.

Network graph of the topmost positively and negatively significantly correlated genes for each PI3K-associated target gene across all cancer cell lines, following Pearson correlation with Benjamini-Hochberg multiple testing adjustment method (*q* < 0.05). Edge weight: absolute *r*-value; edge width: −log_10_(*q*-value); vertex size: weighted vertex degree; network group: component; Fruchterman and Reingold force-directed layout; self-correlations omitted.


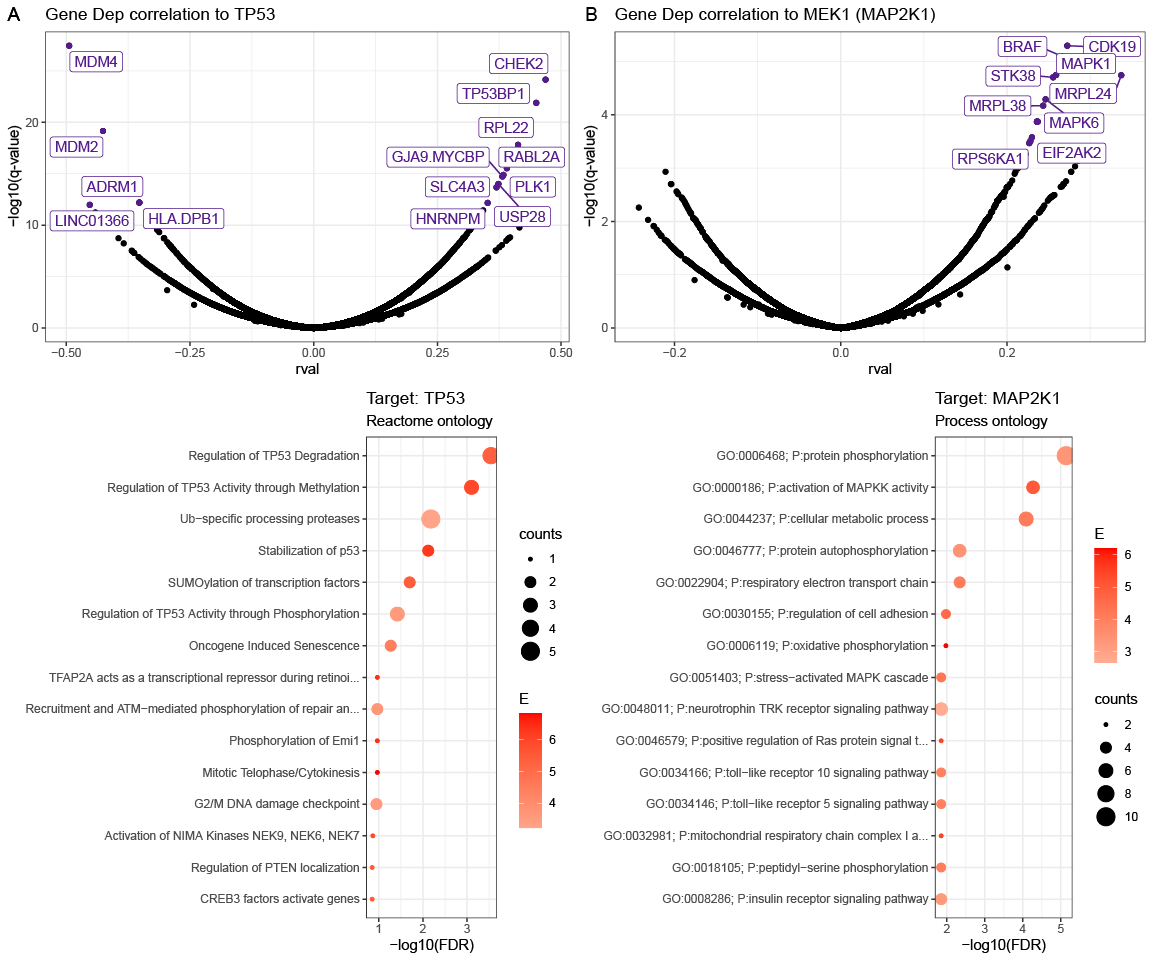


Figure S5. Functional Pathway Inference Analysis identifies components of proliferative signalling pathways.

FPIA was carried out using *TP53* (panel A) or *MAP2K1* (panel B) as target genes. The top correlated genes are highlighted in volcano plots (top) and the top 50 most correlated genes were subjected to over-representation analysis (bottom).


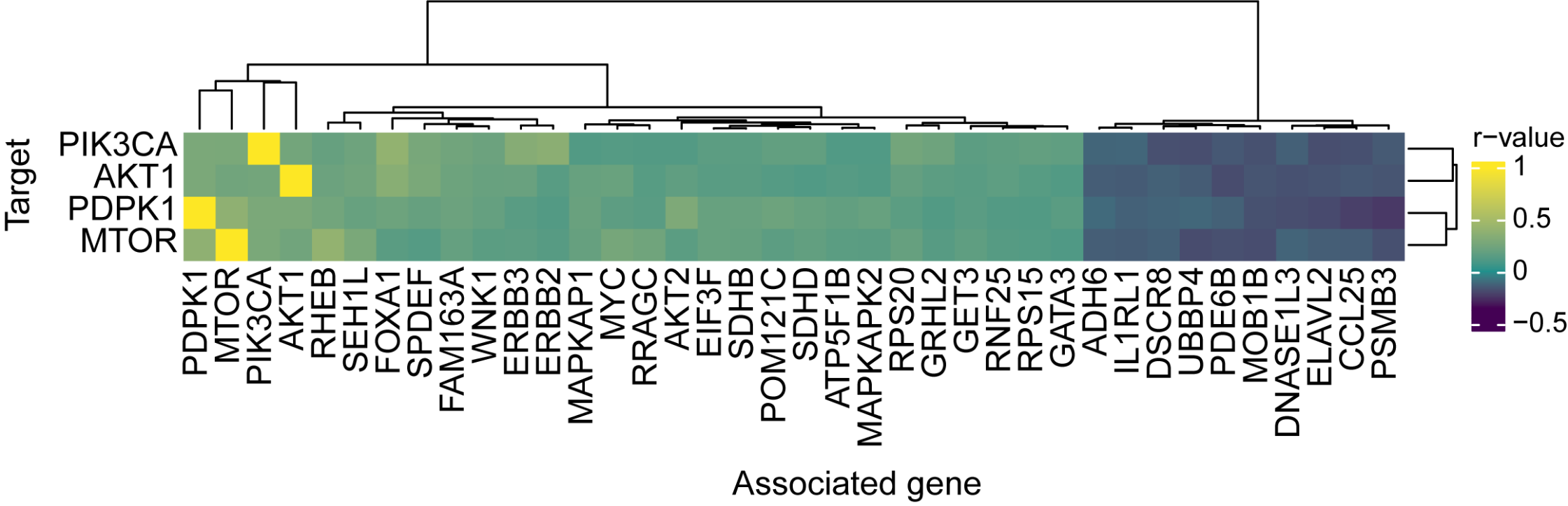


Figure S6. Unsupervised clustering of FPIA results identifies relationships between core PI3K genes.

Hierarchical cluster analysis on a subset of target genes in the canonical PI3K/AKT pathway and the intersection of their shared significant correlations. Hierarchical cluster analysis with Euclidean distances applied to Ward's minimal increase of sum-of-squares agglomeration method. Data presented with self-correlations included.
